# Editorial: Molecular and functional profiling in breast cancer: implications for hereditary and sporadic cases

**DOI:** 10.3389/fonc.2026.1773220

**Published:** 2026-02-09

**Authors:** Alessandra Virga, Michela Cortesi, Sara Bravaccini, Gianluca Tedaldi

**Affiliations:** 1 Biosciences Laboratory, IRCCS Istituto Romagnolo Per Lo Studio Dei Tumori (IRST) “Dino Amadori”, Meldola, Italy; 2 Department of Medicine and Surgery, University of Enna “Kore”, Enna, Italy; 3 Department of Laboratory and Transfusion Medicine, Hub Laboratory, AUSL Romagna, Cesena, Italy

**Keywords:** breast cancer, hereditary breast and ovarian cancer, molecular characterization, next-generation sequencing, functional analysis, sporadic breast cancer

Breast cancer (BC) is a highly heterogeneous disease, encompassing several biological behaviours, clinical trajectories, and therapeutic vulnerabilities. The advent and rapid evolution of molecular profiling have reshaped the landscape of BC research and care, offering unprecedented insight into the mechanisms underlying tumour development, progression, and treatment response. This Research Topic brings together five contributions exploring germline and somatic alterations, functional assays for variant interpretation, tumour microenvironmental features, and the translational implications of genomic discoveries. Collectively, these works illustrate the power of integrated molecular approaches to refine risk assessment, inform therapeutic decision-making, and advance precision oncology.

A key focus of this Topic is the diagnostic and prognostic significance of germline variants in hereditary cancer syndromes (1). In particular, the emerging contribution of non-coding variants to hereditary BC predisposition is becoming increasingly obvious (2). In this context, Dai et al. present a compelling case study characterizing, for the first time, the rare BRCA1 c.5193 + 2dupT variant, identified in a family with a strong history of ovarian cancer. Through functional assays, including minigene-splicing analysis, the authors demonstrate that this variant induces exon 18 skipping and consequent BRCA1 dysfunction. Their work showed how functional validation fills critical gaps when population and genomic data, and in silico predictions are insufficient, ultimately improving variant classification and clinical management of hereditary breast and ovarian cancer.

The importance of germline variation beyond BRCA1/2 genes is further highlighted by Sharaf et al., who examine the APC I1307K missense variant in a Jordanian cohort. Their analysis reveals that this allele, previously well described in Ashkenazi Jewish populations, is also encountered in Arab patients and appears to be associated not only with colorectal but also with BC. These findings underscore the need for population-specific genetic studies and raise important considerations for genetic counselling, surveillance strategies, and risk assessment models in diverse ancestral groups.

Complementing these hereditary perspectives, this Research Topic includes two contributions addressing molecular events that shape tumour behaviour and immune interactions in sporadic BC. Benthami et al. investigate Siglec-7, an inhibitory glycoimmune checkpoint, in BC tissues and publicly available datasets. They demonstrate that Siglec-7 overexpression correlates with aggressive pathological features, immunosuppressive cell infiltration, and signatures of resistance to chemotherapy, endocrine therapy, and immunotherapy. Their work expands current understanding of the sialylation in tumour microenvironment and identifies Siglec-7 as a promising biomarker and potential therapeutic target, particularly in immune-cold subtypes such as triple-negative BC.

A complementary clinical perspective is provided by Zheng et al., who describe a rare presentation of triple-negative breast cancer characterized by symptomatic bone marrow metastasis at the time of diagnosis. While bone involvement is frequent in advanced breast cancer, clinically evident bone marrow infiltration associated with cytopenia is exceedingly uncommon as an early manifestation. In this context, the authors highlight the diagnostic value of bone marrow biopsy in patients with TNBC presenting with unexplained hematologic abnormalities, even when standard imaging fails to demonstrate overt bone marrow involvement. This case underscores the aggressive biological nature of TNBC and illustrates how the integration of clinical findings with pathological and functional assessment can facilitate timely therapeutic intervention and appropriate supportive care.

Finally, Huang et al. contribute a bibliometric analysis of global research trends in the field of DNA damage repair in BC. By mapping publication patterns, influential authors, and emerging themes across decades of research, their work situates the contributions of this Research Topic within broader scientific advances. Their findings reaffirm the centrality of DDR pathways in BC pathogenesis (3) and highlight growing interest in PARP inhibitors, combination therapeutic strategies, the clinical relevance of both germline and somatic DDR alterations, and the emerging challenge of resistance to PARP inhibition (4). Recent real world and genomic studies further support the clinical relevance of HRD signatures and alterations in homologous recombination genes beyond BRCA1/2 in predicting response to PARP inhibitors (5, 6).

Taken together, the articles within this Research Topic illuminate the complex role of molecular profiling across hereditary and sporadic BC. The authors demonstrate how genetic sequencing, functional genomics, immunoprofiling, and computational approaches converge to refine diagnosis, identify actionable vulnerabilities, and enhance the personalization of care. Importantly, they also draw attention to persistent challenges, including the interpretation of rare variants, the integration of molecular findings into clinical practice, and the ethical considerations surrounding genetic testing and informed consent.

Yet the substantial time, cost, and analytical effort required for these advanced approaches raises the question of whether they are truly justified by the potential for better patient treatment or represent an unsustainable burden for resource-limited hospitals. As molecular technologies continue to expand, bridging the gap between genomic discovery and clinical implementation remains a critical priority. The contributions assembled here reinforced the need for collaborative research spanning laboratory sciences, clinical oncology, immunology, bioinformatics, and ethics. We hope that this Research Topic will inspire further interdisciplinary efforts to improve outcomes for individuals affected by BC and to advance the promise of precision medicine.
